# A Novel Peptide-Based SILAC Method to Identify the Posttranslational Modifications Provides Evidence for Unconventional Ubiquitination in the ER-Associated Degradation Pathway

**DOI:** 10.1155/2013/857918

**Published:** 2013-02-03

**Authors:** Veronica G. Anania, Daisy J. Bustos, Jennie R. Lill, Donald S. Kirkpatrick, Laurent Coscoy

**Affiliations:** ^1^Division of Immunology and Pathogenesis, Department of Molecular and Cell Biology, University of California, Berkeley, CA 94720-3200, USA; ^2^Department of Protein Chemistry, Genentech Inc., South San Francisco, CA 94080, USA

## Abstract

The endoplasmic reticulum-associated degradation (ERAD) pathway is responsible for disposing misfolded proteins from the endoplasmic reticulum by inducing their ubiquitination and degradation. Ubiquitination is conventionally observed on lysine residues and has been demonstrated on cysteine residues and protein N-termini. Ubiquitination is fundamental to the ERAD process; however, a mutant T-cell receptor **α** (TCR**α**) lacking lysine residues is targeted for the degradation by the ERAD pathway. We have shown that ubiquitination of lysine-less TCR**α** occurs on internal, non-lysine residues and that the same E3 ligase conjugates ubiquitin to TCR**α** in the presence or absence of lysine residues. Mass-spectrometry indicates that WT-TCR**α** is ubiquitinated on multiple lysine residues. Recent publications have provided indirect evidence that serine and threonine residues may be modified by ubiquitin. Using a novel peptide-based stable isotope labeling in cell culture (SILAC) approach, we show that specific lysine-less TCR**α** peptides become modified. In this study, we demonstrate that it is possible to detect both ester and thioester based ubiquitination events, although the exact linkage on lysine-less TCR**α** remains elusive. These findings demonstrate that SILAC can be used as a tool to identify modified peptides, even those with novel modifications that may not be detected using conventional proteomic work flows or informatics algorithms.

## 1. Introduction

Approximately one-third of newly synthesized proteins are targeted for the degradation by the proteasome within minutes of their synthesis as a result of premature termination or misfolding. ER-associated degradation (ERAD) is a specialized pathway that targets misfolded nascent proteins in the endoplasmic reticulum (ER) for degradation via the proteasome [1]. In the ER, misfolded proteins are recognized by several quality control mechanisms that escort them to a channel for retrotranslocation to the cytosol. Soon after retrotranslocation, these proteins are ubiquitinated and extracted from the ER membrane with the help of the Cdc48p/p97 complex. Ubiquitination acts as a signal to target proteins to the proteasome and thus is an essential process in the degradation of newly synthesized defective proteins.

Ubiquitination is a post-translational modification that regulates many cellular events such as protein degradation, endocytosis, and the cell cycle. The process of ubiquitination involves the concerted action of an E1 ubiquitin-activating enzyme, an E2 ubiquitin-conjugating enzyme, and an E3 ubiquitin ligase. The last step of ubiquitination is mediated by an E3 ubiquitin ligase that provides specificity to the pathway by binding both the target substrate molecule and an E2 ubiquitin-conjugating enzyme (E2) loaded with ubiquitin. Ubiquitin is then transferred from the E2 active site cysteine to a lysine residue of the substrate, forming an isopeptide bond between the substrate and ubiquitin [2]. 

Not all misfolded or defective proteins necessarily encode for or contain accessible lysine residues for ubiquitination, yet these proteins must still be degraded. Thus, it is tempting to speculate that degradation of misfolded or defective proteins relies on a mechanism that is not solely dependent on lysine residues. Interestingly, it was recently reported that proteins may be ubiquitinated on nonlysine residues. For example, N-recognins, a particular class of E3 ligases, have the ability to ubiquitinate the *α*-amino group on the N-terminus of their substrates in order to mediate degradation [3]. More recently, viral E3 ubiquitin ligases encoded by Kaposi's sarcoma-associated herpes virus and its murine homologue, MHV68, have been reported to mediate degradation of surface molecules by promoting their ubiquitination on cysteine and serine/threonine residues, respectively [4, 5]. Several cellular proteins are also known to undergo unconventional ubiquitination, including Pex5p, which is thought to be ubiquitinated on serines/threonines and Bid, which is ubiquitinated on cysteine residues [6, 7]. Given the large number of cellular E3 ubiquitin ligases present (>600 identified [8]), it is unknown whether nonlysine ubiquitination is specific to a few highly specialized E3s or if it is a process common to many E3s that remains thus far underappreciated. Critically though, direct evidence of serine and threonine targeted ubiquitination, in the form of an ester linked −GG signature peptide (ggSP) has remained elusive.

Interestingly, a mutant form of the T-cell receptor *α* subunit (TCR*α*) that lacks lysine residues can be targeted to the proteasome via ERAD through unknown mechanisms [9, 10]. Both lysine and nonlysine ubiquitination of TCR*α* are dependent upon a functional Hrd1 E3 ligase indicating that this E3 ligase can facilitate both lysine and nonlysine ubiquitination. In this study, we use a novel SILAC method coupled with LC-MS/MS to show that several peptides near the C-terminus of lysine-less TCR*α* (KR-TCR*α*) are involved in the association between ubiquitin and KR-TCR*α*, in contrast to wildtype TCR*α* (WT-TCR*α*), where direct modification of lysine residues can be observed. These results strongly suggest that in the absence of lysine residues, the cellular protein degradation machinery has acquired the ability to ubiquitinate nonlysine residues in order to prevent the accumulation of defective peptides. So far, existing bioinformatics tools have been unable to provide direct evidence for unconventional ubiquitination. However, using a novel SILAC approach, we have been able to determine which specific peptide becomes modified, even though the composition of the posttranslational modification(s) remains unknown.

## 2. Materials and Methods

### 2.1. Cell Culture and Reagents

HEK293T cells (number CRL-11268; American Tissue Culture Collection) were maintained in Dulbecco's modified Eagle's medium supplemented with 10% fetal bovine serum and 100 *μ*g/mL penicillin/streptomycin and transfected using Fugene HD (Roche) according to the manufacturer's instructions. Restriction grade thrombin (Novagen) was used according to manufacturer's instructions. PNGase F (NEB) digestions were carried out according to the manufacturer's instructions. Cycloheximide (Sigma) was used at 100 *μ*g/mL for 0, 1, or 2 hours prior to lysis.

### 2.2. Plasmids

pCDNA3.1 vectors expressing an HA tagged version of WT-TCR*α* and KR-TCR*α* were gifts from Dr. Ron Kopito (Stanford University). pCDNA3.1 vectors expressing an myc tagged version of either wildtype Hrd1-mycHis or a dominant negative form of Hrd1 (C240A-) mycHis were gifts from Dr. Emmanuel Wiertz (Leiden University Medical Center, Netherlands). The NE (Neutrophil elastase) construct was provided by Dr. Marshall Horowitz (University of Washington, Seattle, WA) and subcloned into pCDNA3.1 using BamHI and XhoI restriction sites. MSCV HA-HLA-A2 was provided by Dr. Kathleen Collins (University of Michigan Medical School). The HSV-epitope tag (QPELAPEDPED) was inserted downstream of the TCR*α* signal sequence (MQRNLGAVLGIL) using overlap PCR. The thrombin cleavage site (LVPRGS) was cloned downstream of the HSV-epitope tag using a similar method. Threonine 262 and Serine 267/268 mutations to alanine and modified HA tags (YPYDVPDYASL → YPYDVPDYAL) were cloned using PCR site-directed mutagenesis.

### 2.3. Immunoprecipitation, Western Blot Analysis and Antibodies

Cells were transfected 48 hrs prior to cell lysis. Cells were treated with 10 *μ*M MG132 (unless otherwise noted) for 3 hrs and then lysed in RIPA lysis buffer (50 mM Tris-HCl pH 8, 150 mM NaCl, 1% Nonidet P40, 0.5% Sodium Deoxycholate, and 0.1% SDS) supplemented with protease inhibitor cocktail EDTA-free (Roche). Cellular lysates were quantified using the BCA Protein Assay kit (Pierce) according to the manufacturer's protocol. For immunoprecipitation, 1 mg of protein was incubated with 1 *μ*g of anti-HA (Santa Cruz Biotech) antibody and 30 *μ*L protein A/G agarose beads (Santa Cruz Biotech) overnight. Samples were separated on 12% polyacrylamide gels and transferred to PVDF membrane. Blots were probed with either anti-HA (Santa Cruz Biotech), anti-*β*-actin (Abcam), anti-ubiquitin P4D1 (Santa Cruz Biotech), anti-His (Santa Cruz Biotech), or anti-HSV (Genscript Corp.). For double immunoprecipitation, samples were reimmunoprecipitated with 25 *μ*L anti-HSV agarose overnight in RIPA lysis buffer.

*Reverse Transcription Quantitative Real-Time PCR (RT-qPCR).* 293T were transfected with siRNA against Hrd1 (ON-TARGETplus SMARTpool Human SYVN1, Dharmacon) or a negative control siRNA (AllStars Neg. Control siRNA, Qiagen) using Lipofectamine 2000 (Invitrogen) according to the manufacturer's instructions. 24 hours posttransfection, RNA was extracted in Trizol (Invitrogen), treated with RQ1 DNase (Promega), and total RNA was reverse transcribed using oligo (dT) 15 primer (Promega) and SuperScriptII (Invitrogen) at 42°C for 50 minutes. cDNAs were extracted to analyze levels of GAPDH and Hrd1 mRNA using ABI7300 Real-Time PCR System.

### 2.4. Alkaline Hydrolysis

Cells were transfected 48 hrs prior to hydrolysis. Cells were incubated with 10 *μ*M MG132 for 3 hrs and then washed 3 times with 10 mL PBS, once with 10 mL of 100 mM NaCl and then resuspended in 100 mM Na_2_CO_3_ pH 11.5. Samples were dounced 10 times in a 2 mL glass dounce homogenizer (tight pestle) and then incubated on ice for 30 min. Samples were then ultracentrifuged (TLA100.2 rotor, 60 min, 68 K rpm, 4°C). Membrane fractions were recombined with soluble fractions and added to 2X RIPA plus protease inhibitor cocktail. Samples were quantified and immunoprecipitated with the same protocol listed above.

### 2.5. SILAC/Mass Spectrometry

HEK293T cells were cultured in SILAC DMEM (Pierce) lacking lysine and arginine that was supplemented with isotopically enriched forms of L-Leucine (^13^C_6_, 50 *μ*g/mL), (L-lysine (^13^C_6_, ^15^N_2_ hydrochloride; 50 *μ*g/mL), and L-arginine (^13^C_6_, ^15^N_4_ hydrochloride; 40 *μ*g/mL) (heavy) or the corresponding unlabeled form of each at the same concentration (light). Both heavy and light forms of DMEM were further supplemented with L-proline (200 *μ*g/mL; to prevent metabolic conversion of Arg → Pro), L-glutamine (2 mM), and 10% dialyzed FBS (Pierce). After 5 days of metabolic labeling, cells were transfected with the denoted constructs and allowed to express the constructs for 48 hr prior to treatment with MG132 (10 *μ*M, 3 hr). Cells were lysed in RIPA buffer and immunoprecipitated with 2 *μ*g anti-HA antibody and 50 *μ*L protein A/G agarose beads for 3 hours. Immunoprecipitations were washed and TCR*α* was eluted using 30 *μ*L HA peptide (1 mg/mL in TBS) for 30 min at room temperature. Elutions were transferred to clean tubes and digested with PNGase F (New England Biolabs) according to the manufacturer's instructions. Samples were separated by SDS-PAGE and then stained with Simply Blue Coomassie stain (Invitrogen) according to the manufacturer's instructions. Regions of the gel containing unmodified TCR*α* (low molecular weight) and modified TCR*α* (high molecular weight) were excised and diced into 1 mm cubes. Proteins were subjected to overnight in gel trypsin digestion in the presence of 10 ng/*μ*L trypsin resuspended in 50 mM ammonium bicarbonate/5% acetonitrile. Peptides were extracted with 50% acetonitrile/5% formic acid and then dried to completion, resuspended in 4% acetonitrile/5% formic acid/0.01% hydrogen peroxide and then heavy and light samples were combined for analysis. 

For LC-MS/MS analysis, samples were analyzed on an LTQ-Orbitrap XL (ThermoFisher, San Jose, CA) with an ADVANCE electrospray ionization source (Michrom, Auburn, CA). Peptides were loaded onto a 0.1 mm × 100 mm waters 1.7 *μ*m BEH-130 C18 column for 10 min and separated at 1.0 *μ*L/min by a gradient where solvent B (98% acetonitrile/0.1% formic acid) was ramped from 5% to 25% over 35 min and then from 25% to 50% over 2 min. The mass spectrometer was operated such that a duty cycle was comprised of one full MS scan collected at 60000 resolution in the Orbitrap and eight MS/MS scans in the ion trap. For targeted analysis, four of the eight scan events were devoted to ions corresponding to ggSP from KR-TCR*α*. Quantitative data was manually interrogated using QualBrowser v2.07 (ThermoFisher). For peptide identification, MS/MS spectra were searched using Mascot against a concatenated target-decoy database comprised of human protein sequences, known contaminants, TCR*α* sequences of interest, and the reversed versions of each with a 50 ppm precursor ion tolerance and 0.8 Da fragment ion tolerant. Variable modifications were permitted for methionine oxidation (+15.9949 Da) and ubiquitin di-glycine remnant (−GG signature) on lysine, cysteine, serine, or threonine (+114.0429 Da). No more than three variable modifications were permitted in a given search. Precursor ion mass accuracy was used as an orthogonal filter prior to manual inspection of peptide spectral matches.

### 2.6. *In Vitro* Ubiquitination of UbcH5c (E2 Charge Assay)

Reactions (15 *μ*L) of an E2-ubiquitin thioester (or ester for DN) were carried out by combining 0.028 *μ*M E1, 0.5–10 *μ*M E2 (UbcH5c or UbcH5c-DN), and 0.2 *μ*M ubiquitin in a buffer (50 mM Tris-HCl, pH7.5, 50 mM NaCl, 5 mM MgSO_4_, and 5 mM ATP) and incubated for 30 min at 37°C. Samples were boiled for 5 min and separated by SDS-PAGE and stained with Coomassie Blue prior to the analysis by mass spectrometry.

## 3. Results

### 3.1. KR-TCR*α* Is Ubiquitinated and Degraded by the Proteasome

**Figure 1:**
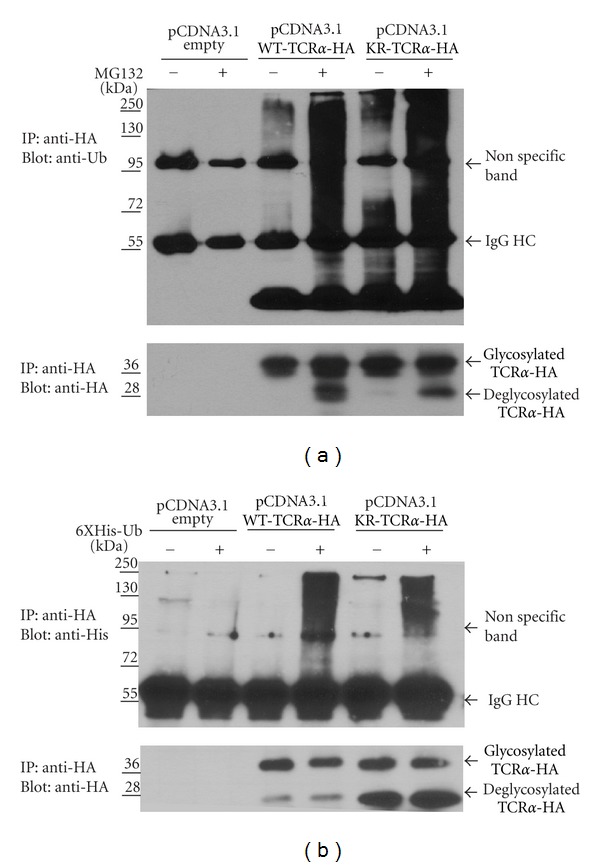
KR-TCR*α* is ubiquitinated and degraded by the proteasome. (a) HEK293T cells were transfected with a vector expressing WT-TCR*α*, or a vector expressing KR-TCR*α*. Cells were either untreated or incubated with MG132 before lysis. Cellular lysates were immunoprecipitated using an anti-HA antibody and the ubiquitination status of TCR*α* was analyzed by Western blot analysis using an anti-ubiquitin antibody (top panel). Membranes were stripped and reanalyzed using an anti-HA antibody (bottom panel). (b) HEK293T cells were cotransfected with 6XHis-Ub and either a vector expressing WT-TCR*α* or KR-TCR*α*. Cells were either untreated or incubated with MG132 before lysis. Cellular lysates were immunoprecipitated using an anti-HA antibody and the ubiquitination status of TCR*α* was analyzed by Western blot analysis using an anti-His antibody (top panel). Membranes were stripped and reanalyzed using an anti-HA antibody (bottom panel). Molecular weight markers (kD) of marker proteins are indicated.

In order to understand the molecular mechanism allowing for lysine-less proteins to be targeted by the ERAD pathway, we employed KR-TCR*α* as a substrate. TCR*α* is an ideal candidate protein to study ERAD because in non-T cells, TCR*α* lacks binding partners like CD3 and TCR*β*, and therefore the vast majority is misfolded and targeted through the ERAD pathway. HEK293T cells were transiently transfected with an empty vector control, a vector encoding HA-tagged WT-TCR*α* or HA-tagged mutant TCR*α* (KR-TCR*α*) in which all available lysines were mutated to arginines. Cells were either untreated or treated for 3 hours with the proteasome inhibitor MG132. Cellular lysates were subjected to immunoprecipitation using an anti-HA antibody, and the presence of ubiquitinated TCR*α* was examined by the Western blot analysis. As shown in Figure 1(a), MG132 treatment increased the level of polyubiquitination on both WT-TCR*α* and KR-TCR*α* compared to untreated samples.

To verify the specificity of the anti-ubiquitin antibody, HEK293T cells were cotransfected with a vector encoding 6XHis-tagged ubiquitin (6XHis-Ub) together with a vector containing HA-tagged WT-TCR*α* or KR-TCR*α*. Cellular lysates were immunoprecipitated with an anti-HA antibody, and 6XHis-ubiquitin incorporation into high molecular weight species of TCR*α* was visualized by Western blot using an anti-His antibody.  Figure 1(b) shows that 6xHis-Ubiquitin is indeed incorporated into both WT-TCR*α* and KR-TCR*α* high molecular weight species indicating that both are extensively poly-ubiquitinated. Western blots were stripped and reprobed with an anti-HA antibody to verify all constructs were expressed at comparable levels (Figures 1(a) and 1(b)). MG132 treatment revealed an additional low molecular weight species which has been shown to represent a deglycosylated version of TCR*α* that has been extracted from the ER membrane [11]. In the absence of MG132, deglycosylated TCR*α* is rapidly degraded. The observed stabilization of deglycosylated KR-TCR*α* by MG132 strongly suggests that similar to WT-TCR*α*, KR-TCR*α* is degraded in a proteasome-dependent manner.

It is possible that the high molecular weight ubiquitin signal observed could represent an unknown KR-TCR*α*-associated cofactor that is ubiquitinated and required for targeting of KR-TCR*α* to the proteasome. In order to address the possibility that the observed signal may be due to ubiquitination of a sacrificial protein that coimmunoprecipitates with TCR*α*, we performed a double immunoprecipitation experiment. Figure S1 shows that even after TCR*α* immunoprecipitations have been completely denatured and reimmunoprecipitated (Supplementary Figures are available online at http://dx.doi.org/10.1155/2013/857918); we still observe robust ubiquitination of both WT-TCR*α* and KR-TCR*α*. This result suggests that both proteins are physically associated with ubiquitin and, consequently, that KR-TCR*α* is directly ubiquitinated.

N-terminal ubiquitination has been described for several proteins and is lysine-independent. To determine whether KR-TCR*α* is ubiquitinated on its N-terminus, an HSV tag followed by a thrombin cleavage site (TMB) was introduced downstream of the TCR*α* signal peptide in the WT-TCR*α* or KR-TCR*α* backbone (Figure S2A). Cellular lysates were immunoprecipitated with an anti-HA antibody, and the resulting immunoprecipitates were digested with thrombin and washed extensively to eliminate N-terminal peptides. As shown in Figure S2B, ubiquitination was readily detectable with the various TCR*α* constructs, regardless of whether the N-terminus was present. These results are consistent with ubiquitination of KR-TCR*α* on internal, nonlysine residues.

### 3.2. The E3 Ubiquitin Ligase Hrd1 Is Involved in the Ubiquitination of Both WT-TCR*α* and KR-TCR*α*

**Figure 2:**
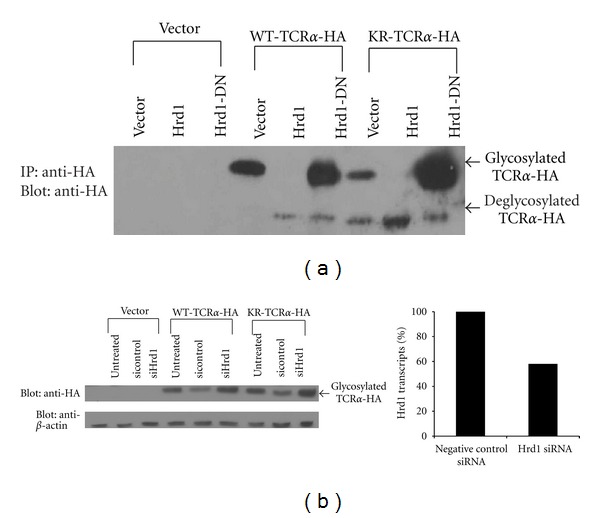
Degradation of WT-TCR*α* and KR-TCR*α* is dependent upon the E3 ubiquitin ligase Hrd1. (a) The various TCR*α* constructs were cotransfected with WT-Hrd1 or DN-Hrd1. Transfected cells were incubated with MG132 before lysis. Cellular lysates were immunoprecipitated with an anti-HA antibody and analyzed by Western blot using an anti-HA antibody. (b) 293T were cotransfected with a SMARTPool of siRNA against Hrd1 or negative control siRNA and vector, WT-TCR*α*, or KR-TCR*α* (HA-tagged). 24 hrs posttransfection, cells were treated with MG132 and lysed and levels of TCR*α* were determined by Western blotting with an anti-HA antibody. Levels of Hrd1 were determined by qRT-PCR.

Ubiquitination of WT-TCR*α* is known to require Hrd1 [12, 13], an E3 ubiquitin ligase of the RING finger family that mediates degradation of many ER substrates [1]. To determine whether Hrd1 is also required for the degradation of KR-TCR*α*, HEK293T cells were cotransfected with expression vectors for the different TCR*α* constructs together with vectors encoding wildtype Hrd1 (Hrd1-WT) or a dominant negative version of Hrd1 (Hrd1-DN) [12]. Degradation of TCR*α* was assessed by Western blot analysis. As shown in Figure 2(a), the expression of Hrd1-DN is associated with an accumulation of the glycosylated form and a decrease in the deglycosylated form of both WT-TCR*α* and KR-TCR*α*. By contrast, the expression of Hrd1-WT led to a complete loss of the glycosylated forms of both WT-TCR*α* and KR-TCR*α*. Additionally, siRNA knockdown of Hrd1 led to an accumulation of glycosylated forms of both WT-TCR*α* and KR-TCR*α* (Figure 2(b)) relative to a nontargeting control siRNA. Altogether, these results indicate that Hrd1 mediates KR-TCR*α* retrotranslocation and strongly suggest that Hrd1 is the E3 ubiquitin ligase responsible for degradation of both WT-TCR*α* and KR-TCR*α*. These results are in agreement with recent data showing that Hrd1 is also required for ubiquitination of a lysine-less version of NS-1, another model ERAD substrate [13].

### 3.3. KR-TCR*α* Ubiquitination Is Partially Sensitive to Mild Alkaline Hydrolysis

Ubiquitination on cysteine, serine, and threonine residues has recently been reported [4–7, 13, 14]. Cysteine ubiquitination involves the formation of thioester bonds, which are sensitive to reducing conditions. Because experiments illustrated in Figures 1, S1, and S2 were performed in the presence of the reducing agent *β*2-mercaptoethanol, it is unlikely that cysteine ubiquitination is contributing to the ubiquitin signal observed on KR-TCR*α*. Ubiquitination of serine and threonine residues would presumably occur via ester bonds that are not reduced under standard denaturing conditions, but would be susceptible to mild alkaline hydrolysis. Alkaline hydrolysis treatment does not affect isopeptide bonds, which are formed by ubiquitination of lysine residues. To test if KR-TCR*α* associated ubiquitin may reside on serine or threonine residues, HEK293T cells were transiently transfected with WT-TCR*α*, KR-TCR*α*, or a vector control and were treated with MG132 to allow for the accumulation of ubiquitinated TCR*α* proteins. Cells were then lysed and subjected to mild alkaline hydrolysis for two hours. Cellular lysates were immunoprecipitated using an anti-HA antibody and analyzed by Western blot using an anti-ubiquitin antibody. The level of ubiquitination in hydrolyzed samples was quantified relative to unhydrolyzed samples using densitometry (Figure S3B). Interestingly, ubiquitination of WT-TCR*α* is not sensitive to alkaline hydrolysis, which suggests that when lysine residues are present, they are the preferred targets of ubiquitin. By contrast, ubiquitination of KR-TCR*α* is partially diminished upon mild alkaline treatment (Figure S3A), despite the quantity of unmodified TCR*α* being similar in all samples (Figure S3B). This level of hydrolysis is in accordance with what other reports have demonstrated for serine/threonine ubiquitination [5, 7, 13]. Harsher alkaline treatments were investigated; however, this led to degradation of the unmodified protein for both WT-TCR*α* and KR-TCR*α* (data not shown). Presumably, if ubiquitination on KR-TCR*α* was solely attached via an ester linkage, mild alkaline treatment should lead to complete hydrolysis of this bond. This result suggests that KR-TCR*α* may be ubiquitinated on nonlysine residues via ester linkages in addition to other unknown linkages, or by a linkage that is only partially susceptible to alkaline hydrolysis.

### 3.4. WT-TCR*α* Is Ubiquitinated on Lys-118, -144, and -178

**Figure 3:**
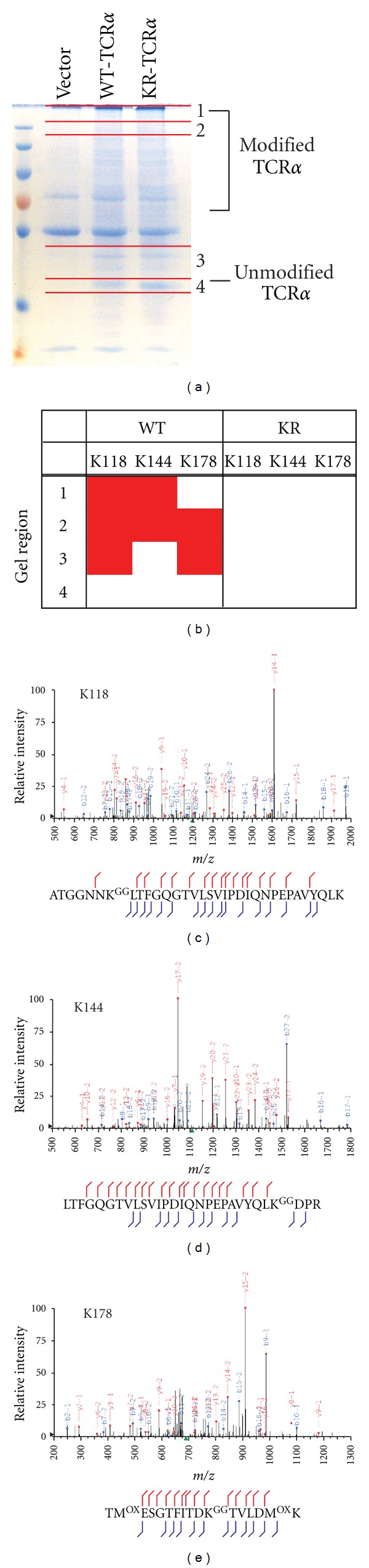
Tandem mass-spectrometry identification of −GG signature peptides from ubiquitinated TCR*α* on Lys-118, Lys-144, and Lys-178. HEK293T cells were transiently transfected with WT-TCR*α* or vector alone, incubated with MG132, and then lysed. Lysates were subjected to immunoprecipitation with an anti-HA antibody and examined by SDS-PAGE followed by Coomassie staining. Four regions of the gel were excised and trypsinized as shown in (a). Samples were analyzed by tandem mass-spectrometry and the results are summarized in (b). Peptide spectral matches to −GG signature peptides (ggSP) at Lys-118, Lys-144, or Lys-178 in each region of the gel are indicated by red shading. Representative MS/MS spectra for each of the ggSP are shown: (c) Lys-118, (d) Lys-144, and (e) Lys-178. The peaks are labeled with the fragment ion and its charge state. For example, y14-1 is the singly charged version of y14. b- and y-fragment ions are denoted by blue and red, respectively. Green squares denote precursor ions, multiply charged fragment ions, or predictable neutral loss ions accounted for during manual inspection of MS/MS data.

To determine which residues on WT-TCR*α* could be modified by ubiquitin, samples containing ubiquitinated WT-TCR*α* were subjected to LC-MS/MS analysis. HEK293T cells were transfected with TCR*α* or vector alone, incubated with MG132 and then lysed. Lysates were subjected to immunoprecipitation with an anti-HA antibody and examined by SDS-PAGE followed by Coomassie Blue staining (Figure 3(a)). Four separate regions (labeled 1–4, Figure 3(a)) were excised for each sample and subjected to in-gel trypsin digestion. Trypsinization of an ubiquitinated protein yields a diglycine signature as a remnant at the position where ubiquitin was bound and results in a mass increase of 114.0429 Da on ggSP. Samples were analyzed by LC-MS/MS, and the resulting spectra searched for ggSP using the Mascot search algorithm. Of the twelve lysine-containing peptides of WT-TCR*α*, ubiquitination of Lys-118, Lys-144, and Lys-178 were detected (Figures 3(c)–3(e)). Data collected from digested WT-TCR*α* samples were subsequently searched for the presence of ggSP where serine/threonine modifications were permitted, but none were identified. This data supports the hypothesis that when lysines are accessible, they are the preferred sites of ubiquitination. Similar LC-MS/MS analyses were also performed in an attempt to ascertain the sites of ubiquitination on KR-TCR*α*. Database searches for ggSP with modification of serine/threonine residues again did not yield any modified peptide spectral matches.

### 3.5. Novel SILAC Method Indicates That KR-TCR*α* Is Directly Modified on the Peptide VAGFNLLMTLR

**Figure 4:**
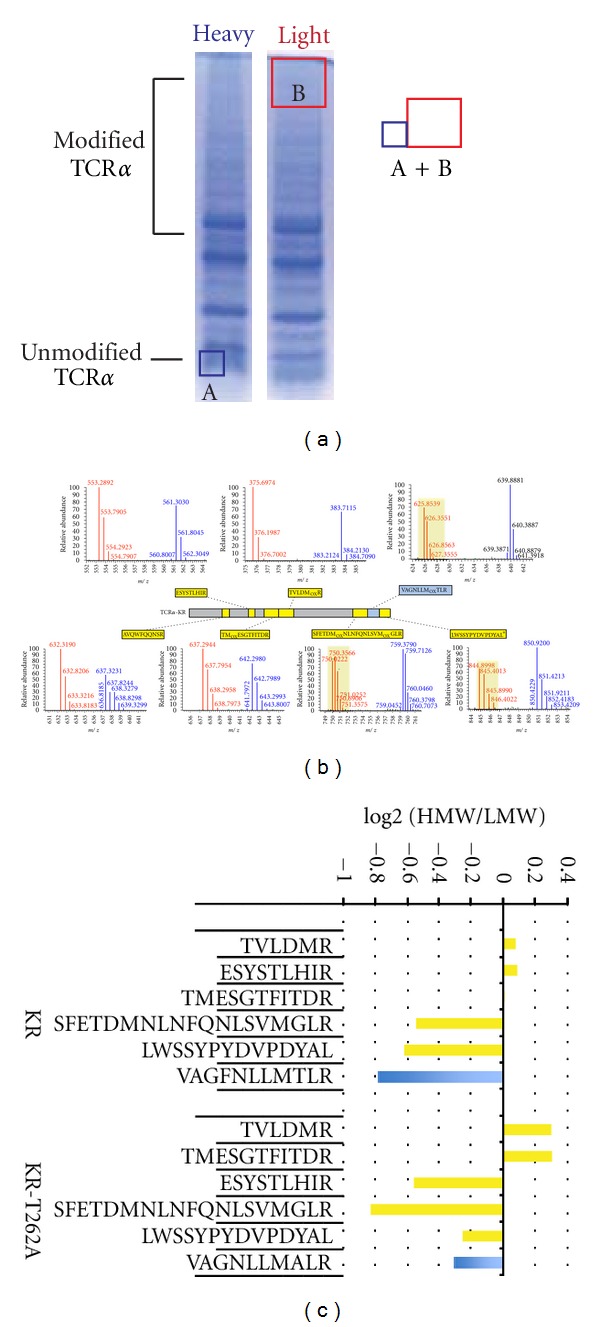
KR-TCR*α* peptide VAGFNLLMTLR (aa 254–264) is modified. HEK293T cells were incubated in either heavy or light SILAC media for five days and then transiently transfected with KR-TCR*α* or vector alone, incubated with MG132 and then lysed. Lysates were subjected to immunoprecipitation with an anti-HA antibody and examined by SDS-PAGE followed by Coomassie staining. (a) Gel pieces containing heavy, unmodified TCR*α* were combined with gel pieces containing light SILAC labeled, ubiquitin-modified KR-TCR*α*. Heavy and light proteins were digested with trypsin and analyzed by LC-MS/MS. (b) Seven narrow range full-MS spectra for representative KR-TCR*α* peptides are shown. Data were collected in high-resolution on an LTQ-Orbitrap, with light ions shown in red and heavy ions in blue. (c) HEK293T cells were incubated in either heavy or light SILAC media for five days, transfected with KR-TCR*α* or KR-TCR*α*-T262A, and the same experimental procedure was followed as in Table 1. The relative abundance of six peptides are quantified and represented in the bar graph. Numbers represent the area under the curve for the peptide in the high molecular weight region (corresponding to modified TCR*α*) divided by the peak height of the same peptide in the low molecular weight region (corresponding to unmodified TCR*α*).

There are twenty-eight serine residues and twenty-five threonine residues present in KR-TCR*α*. Because such a substantial portion of KR-TCR*α* is composed of serine or threonine residues, combinatorial mutagenesis of these residues would most likely prevent entry into the ER which may cause unpredictable changes in the mechanisms of degradation. Therefore, we used stable isotope labeling in cell culture coupled with tandem mass spectrometry (SILAC-MS) to generate evidence for ubiquitin modification on Ser/Thr residues of KR-TCR*α*. Theoretically, the unmodified versions of ubiquitin-modified peptides would be diminished in high molecular weight forms of Ub-KR-TCR*α* relative to unmodified KR-TCR*α*. HEK293T cells were cultured in media supplemented with either the heavy (^13^C_6_,^15^N_2_-Lys, ^13^C_6_,^15^N_4_-Arg, ^13^C_6_-Leu) or light forms of lysine, arginine, and leucine and subsequently transfected with either empty vector or a vector expressing KR-TCR*α*. Cells were treated with MG132, lysed, and then subjected to separate immunoprecipitations with an anti-HA antibody. The unmodified and Ub-KR-TCR*α* in each immunoprecipitation were separated by SDS-PAGE in individual lanes, then stained with Coomassie Blue. Gel regions containing isotopically light, Ub-KR-TCR*α* (high molecular weight) and isotopically heavy, unmodified KR-TCR*α* (low molecular weight) were excised as separate gel bands and digested (Figure 4(a)). After extracting tryptic peptides, a portion of the low molecular weight unmodified KR-TCR*α* (heavy) sample was mixed with peptides from the high molecular weight gel piece containing Ub-KR-TCR*α* (light). LC-MS/MS analysis was performed to determine the relative abundance of heavy and light peptides, with the prediction that heavy to light ratios would be consistent for peptides derived from unmodified regions of sequence. Alternatively, the modification of a residue by ubiquitin would decrease the relative abundance of the corresponding unmodified peptide in the high molecular weight (ubiquitinated) sample relative to its unmodified counterpart in the low molecular weight region. Peptides from the N-terminal portion of the protein were identified and found to have consistent ratios between high and low molecular weight regions. However, for peptides in the C-terminal portion of the protein, a marked decrease was observed in the high molecular weight region. This was particularly apparent for peptides SFETDMNLNFQNLSVMGLR (aa 230–248), VAGFNLLMTLR (aa 254–264), and LWSSYPYDVPDYAL (aa 265–279) (Table 1 and Figure 4(b)). This result suggests that modifications occurring on residues within these sequences are decreasing the abundance of the corresponding peptides. Notably, each of these peptides contains at least one Ser or Thr residue. It is important to note that since we are using a C-terminal HA tag to immunoprecipitate these proteins, these peptides are not dropping out of the high molecular weight fraction because of C-terminal degradation or incomplete translation.

Multiple approaches were carried out in an attempt to match a spectrum from the ubiquitin modified sequences. Neither error tolerant searches in Mascot nor the InsPecT algorithm [15] yielded a hint as to the nature of the modification. Next we carried out targeted LC-MS/MS analysis on the peptide VAGFNLLMTLR (residues 254–264) in hopes of generating a direct evidence of a ggSP in this region. The VAGFNLLMTLR peptide is ideally suited for targeted analysis because it contains a single threonine residue, no serine residues, and showed decreased relative abundance in ubiquitin modified KR-TCR*α*. In the VAGNLLMTLR peptide, it was presumed that the decreased SILAC ratio of unmodified VAGNLLMTLR should coincide with the formation of an ester-linked ggSP of Thr-262. Focused MS/MS scan events targeting the unmodified and ubiquitin modified form of this sequence (+114.0429 Da) were established towards ions in multiple charge states (*z* = 2, *z* = 3). To ensure that methionine oxidation did not confound the analysis, methionine residues were converted into the methionine-sulfoxide form using an optimized hydrogen peroxide/formic acid incubation method recently optimized in our lab [16]. Surprisingly, no spectra were identified as matching to an ester-linked ggSP for the sequence covering Thr-262. To determine if a serine in place of Thr-262 could facilitate dropout of the unmodified peptide signal from the high molecular weight fraction, we mutated Thr-262 to a serine. While KR-TCR*α*-T262S could facilitate dropout from the high molecular weight region, again, no ester-linked ggSP were observed in the targeted analyses (data not shown).

Since we were unable to generate direct evidence for ester-linked ggSP on VAGNLLMTLR, we next tried to determine if Thr-262 is required for dropout of signal of the unmodified version in the high molecular weight fraction of the gel. Thr-262 mutation to an alanine eliminates the possibility of forming an ester-linked ubiquitin. SILAC labeled HEK293T cells transfected with vectors expressing KR-TCR*α* or KR-TCR*α*-T262A were prepared as above. A bar graph representing the relative abundance of the VAGNLLMTLR peptide and five additional KR-TCR*α* peptides are shown in Figure 4(c) to allow comparison of peptides in the high molecular weight gel region B (red) against the same peptides from the low molecular weight, unmodified TCR*α* band (blue). Mutation of Thr 262 to an alanine partially reversed the negative log 2 light/heavy ratio for the peptide VAGFNLLMAR relative to its threonine-containing equivalent, suggesting that in the absence of a threonine (Figure 4(c)), this peptide cannot be modified. Notably though, the T262A mutation did not completely reverse the heavy to light ratio of this peptide relative to peptides from other portions of the sequence. One potential explanation may be that ubiquitin modification of the flanking peptides directly downstream of VAGFNLLMTLR impairs the ability of trypsin to liberate unmodified VAGFNLLMTLR peptide from the TCR*α* sequence. Alternatively, additional posttranslational modifications on this peptide could be preventing positive identification.

### 3.6. Evidence That Ester-Linked and Thioester-Linked Ubiquitin Can Be Detected Using Traditional LC-MS/MS Methods

**Figure 5:**
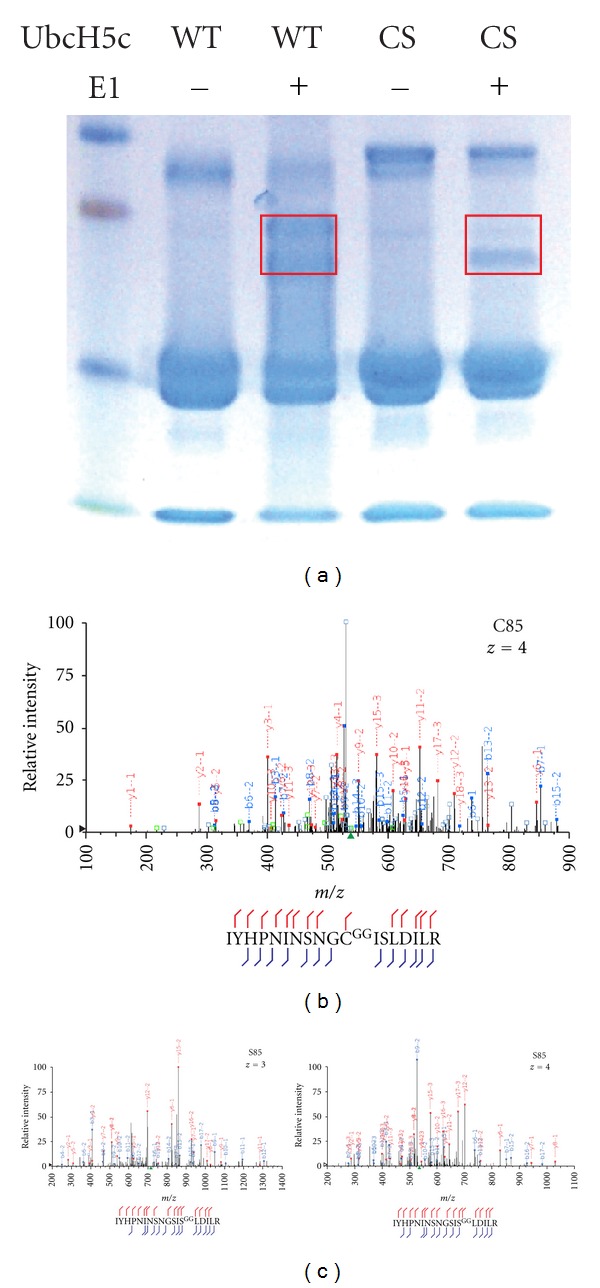
Tandem mass-spectrometry identification of −GG signature on active site serine based on peptide spectral matches from multiple charge states. *In vitro* E2 charging assays were performed on wildtype UbcH5c or C85S-mutant UbcH5c where the catalytic cysteine has been mutated to a serine residue to stabilize its interaction with bound ubiquitin. Ubiquitin-UbcH5c thioester (wildtype) and ester linked ubiquitin-C85S-UbcH5c (CS) were separated by SDS-PAGE and stained with Coomassie Blue (a). Regions denoted in red were excised from the gel, digested, and analyzed by LC-MS/MS. Peptide spectral matches for serine linked ggSP were identified for UbcH5c wild type (b) and for C85S-UbcH5c (c). Representative MS/MS spectra for several charge states are shown: *z* = 3 (right panel) and *z* = 4 (left panel). b- and y-fragment ions are denoted by blue and red, respectively. Green squares denote precursor ions, multiply charged fragment ions or predictable neutral loss ions accounted for during the manual inspection of MS/MS data.

One possible explanation for our inability to identify ggSP on serine, threonine, or cysteine containing peptides could be that our methods of sample preparation followed by LC-MS/MS are too harsh to preserve a thioester or ester bond. In order to determine if this was indeed problematic, we employed an E2 charge assay to conjugate ubiquitin to the E2 ubiquitin conjugation enzyme UbcH5c *in vitro*. Either wildtype UbcH5c or a mutant version (UbcH5c-CS) that has the catalytic cysteine (Cys-85) mutated to a serine was used in the assay. *In vitro*, Ser-85 of UbcH5c-CS will accept ubiquitin and has been used in structural studies to permit the crystallization of an E2 charged with ubiquitin [17]. E2 charge assay reactions were separated by SDS-PAGE and stained with Coomassie Blue. Bands corresponding to charged (ubiquitinated) forms of UbcH5c were excised and subjected to in-gel trypsinization followed by LC-MS/MS (Figure 5(a)). Figures 5(b) and 5(c) show that both thioester- and ester-linked ubiquitin can be detected quite well by our mass spectrometer and also withstands standard LC-MS/MS sample preparation methods. Based on this finding, we conclude that if ubiquitin were linked to KR-TCR*α* via an ester or thioester linkage, we can deliver a suitable peptide to the mass spectrometer for identification.

### 3.7. An Endogenous Protein Naturally Devoid of Lysine Residues Is Ubiquitinated and Degraded in a Proteasome-Dependent Manner

An analysis of the annotated human proteome revealed roughly a dozen validated proteins that are naturally devoid of lysines. One protein, neutrophil elastase (NE), was of particular interest because mutations known to exist within the human population lead to severe congenital neutropenia (SCN). Interestingly, these mutations have been shown to induce the unfolded protein response (UPR) and the level of UPR activation is correlated with the clinical phenotype [18]. HEK293T cells were transiently transfected with a vector control, an HA-tagged version of NE, or an HA-tagged version of HLA-A2, a class I major histocompatibility (MHC-I) molecule, as a control. The ubiquitination status of NE and HLA-A2 was analyzed by Western blot analysis. As shown in the top panel of Figure S4, treatment with MG132 resulted in an accumulation of polyubiquitinated NE, but not HLA-A2, even though both proteins are expressed at similar levels in cellular lysates (Figure S4, bottom panel). This result suggests that NE is ubiquitinated and degraded in a proteasome-dependent manner despite lacking lysine residues. Endogenous proteins are likely undergoing the same posttranslational modifications as KR-TCR*α*; therefore, it is imperative that we better understand this process.

## 4. Discussion

In this study, we have shown that a lysine-less substrate of ERAD is directly ubiquitinated and degraded in a proteasome-dependent manner. This ubiquitination is partially sensitive to mild-alkaline hydrolysis, consistent with the possibility that ubiquitin is attached to serine and threonine residues. Although traditionally used to detect global changes in protein expression, we employed SILAC to identify regions of a single protein that are posttranslationally modified (Figure 4(a)). Using this approach, we show that unmodified forms of the KR-TCR*α* peptides SFETDMNLNFQNLSVMGLR (aa 230–248), VAGFNLLMTLR (aa 254–264), and LWSSYPYDVPDYAL (aa 265–279) are depleted relative to other KR-TCR*α* peptides in high molecular weight Ub-KR-TCR*α*. With only a single Thr residue, and no Lys, Cys, or Ser, it suggests that Thr-262 within VAGFNLLMTLR may indeed carry ubiquitin. In a sample where lysine residues are available for ubiquitin modification, we observe that WT-TCR*α* is directly ubiquitinated on at least three lysine residues within the ectodomain, supporting previous studies which suggest that lysine residues are preferred over nonlysine residues [19]. Our data support a model in which the cellular protein degradation machinery has evolved to ubiquitinate nonlysine residues in cases where lysine residues may not be available, as a means to prevent the accumulation of misfolded or defective proteins.

The existence of alternative modes of ubiquitination in these cellular processes may be essential to ensure the degradation of a highly diverse array of substrates. In support of this, we have shown that the endogenous protein NE, which lacks lysine residues, can also be ubiquitinated and degraded in a proteasome-dependent manner. Although ERAD and cellular quality control systems are highly efficient at eliminating misfolded proteins, the accumulation of defective proteins is observed in numerous diseases such as cystic fibrosis or Parkinson's disease [20]. Similarly, humans that carry genetic mutations in NE develop SCN which is characterized by recurrent bacterial infections and predisposes patients to myelodysplasia and acute myelogenous leukemia [21]. Interestingly, these same genetic mutations in NE have been shown to activate the unfolded protein response and lead to cellular apoptosis [18]. Therefore, it is essential that we better understand the cellular pathways involved in attaching and removing this modification as well as defining its exact chemical composition.

Ubiquitination of substrates has long been thought to occur exclusively through amine groups on lysine residues or the N-terminus of a protein. Recent reports that lysine-less substrates can be ubiquitinated on cysteine, serine, or threonine residues were unexpected. Because such modifications have been observed in only a discrete number of substrates, one could argue that lysine-independent ubiquitination is restricted to a few specialized E3 ligases and a limited number of substrates. Here we show that lysine-independent ubiquitination can occur during ERAD, a process aimed at broadly targeting defective polypeptides within the secretory pathway. This supports a recent report by Shimizu et al. that shows the targeting of NS-1 for ERAD by nonlysine ubiquitination. In the case of NS-1, it was proposed that serine and threonine residues mediate ubiquitin-dependent proteolysis even though the protein contains lysine residues [13]. In our system, we observe that when present, lysines residues are the preferred targets of Hrd1-mediated ubiquitination, while in the absence of lysine residues, Hrd1 is capable of conjugating ubiquitin to nonlysine residues. Perhaps NS-1 and TCR*α* utilize different ERAD components that influence which residues are accessible for ubiquitination. Future studies will determine the regulators of lysine residue accessibility and whether other factors are involved in catalyzing the transfer of ubiquitin to nonlysine residues. It is also possible that a large number of E3 ligases have the potential to perform both lysine and lysine-independent ubiquitination and that a mechanism for the latter has been perhaps unappreciated due to the preponderance of ubiquitination on lysine residues. One lingering concern though is that despite multiple published reports suggesting ubiquitination on Ser and Thr residues, including the current work, direct MS/MS evidence of this phenomenon in the form of a ggSP is lacking. This absence of evidence is conspicuous given that careful studies looking for ggSP with modified Ser/Thr residues have been performed by us and others [13]. Furthermore, we show here that peptides bearing a −GG signature on Ser can be identified and easily detected by traditional LC-MS/MS approaches (Figure 5).

In ERAD, the protein region ubiquitinated varies depending on the substrate and the E3 ubiquitin ligase involved. MK3 is a virally encoded E3 ligase that targets MHC-I proteins for the degradation from the ER. Recent data indicate that ubiquitination of the intracytoplasmic domain of MHC-I on either lysine, serine, or threonine is essential for this process [5]. The only residues present in or proximal to the intra-cytoplasmic domain of KR-TCR*α* that may act as primary ubiquitination sites are Thr-262, Ser-267, and Ser-268. A triple mutant of KR-TCR*α* where Thr-262, Ser-267, and Ser-268 have all been mutated to alanines was also found to be ubiquitinated to the same extent as KR-TCR*α* (Figure S5). Additionally, targeted analysis focusing on ggSP for these three Ser/Thr in KR-TCR*α* did not yield any peptide spectral matches. Since these residues are unnecessary for KR-TCR*α* ubiquitination, our data suggest that perhaps in the absence of these residues, the luminal and transmembrane domains can serve as sites of ubiquitination for KR-TCR*α*. This result demonstrates that the ERAD machinery is quite versatile and given the challenge of a “difficult” substrate still manages to ubiquitinate and degrade misfolded proteins.

Attempts to conclusively characterize the type of bond formed between a serine or threonine residue and ubiquitin have proved to be challenging in this study and elsewhere. Given the large number of possible ubiquitin acceptor sites, the potential for distributed multiubiquitination is quite high. Furthermore, there is a strong probability that alternative residues compensate when primary ubiquitination sites are rendered unusable by site-directed mutagenesis. With that said, ubiquitination of KR-TCR*α* is not completely eliminated by alkaline hydrolysis (Figure S3). While it is formally possible that structural features somehow protect these linkages from hydrolysis, the result of this experiment combined with the absence of evidence for a Ser/Thr linked ggSP argues that the ubiquitin linkage to TCR*α* may not actually be solely through the predicted ester linkage. Of particular concern is that despite SILAC evidence indicating that the majority of VAGFNLLMTLR is modified, no ggSP were detectable for Thr-262 in KR-TCR*α*. It remains possible that ubiquitin is bound to Thr-262 through another partially hydrolyzable bond. Another possibility remains that a secondary “carrier” of ubiquitin is covalently bound to KR-TCR*α* via threonine and that this carrier-KR-TCR*α* bond is itself partially susceptible to alkaline hydrolysis. Whether this “carrier” exists and, moreover, whether it is proteinaceous or not remains to be determined. Examination of identified proteins between vector transfected cells and cells expressing KR-TCR*α* did not reveal any obvious candidates, although additional studies are required. It is potentially noteworthy that Ub-like proteins in the autophagy pathway have been shown to conjugate membrane bound lipids directly [22]. Lastly, this peptide may be additionally modified on another amino acid confounding our ability to identify the ubiquitinated form. Defining the exact chemical composition of nonlysine ubiquitination may require a more refined substrate and/or new algorithms designed to search for peptides with unexpected mass modifications.

In conclusion, we have shown that Hrd1 can ubiquitinate and degrade ERAD substrates using both conventional, lysine ubiquitination and unconventional, lysine-independent ubiquitination. Using SILAC, we have been able to determine which lysine-less peptides are modified, even though the nature of the ubiquitin modification is unknown. This technology could easily be applied to other posttranslational modifications, especially in cases where the chemical composition is unclear or when multiple modifications on a single peptide may be interfering with traditional database search identifications.

## Supplementary Material

The supplemental figures included with this manuscript are biochemical analyses that demonstrate that KR-TCR*α* is modified on an internal, non-lysine residue. In addition, also included is a figure demonstrating that an endogenous, human protein is also ubiquitinated and degraded by the proteasome.

## Figures and Tables

**Table 1:** The normalized light to heavy ratios, denoting the relative abundance of selected peptides, between high molecular weight regions of the gel (HMW: containing light ubiquitin-modified KR-TCR*α*) and low-molecular weight regions of the gel (LMW: containing heavy unmodified KR-TCR*α*). These results are representative of two independent experiments.

KR-TCRa peptides	log⁡2 (HMW/LMW)
R.GDQVEQSPSALSLHEGTGSALR.C	1.009
R.AVQWFQQNSR.G	0.388
R.LTFGQGTVLSVIPDIQNPEPAVYQLR.D	0.242
R.ESYSTLHIR.D	0.087
R.TVLDM^*∧*^R.A	0.074
R.TM^*∧*^ESGTFITDR.T	0.008
R.SFETDM^*∧*^NLNFQNLSVM^*∧*^GLR.I	−0.536
R.LWSSYPYDVPDYAL.-	−0.618
R.VAGFNLLM^*∧*^TLR.L	−0.780

^*∧*^Indicates oxidized methionine.
