# Supplementary material for: A Novel Peptide-Based SILAC Method to Identify the Posttranslational Modifications Provides Evidence for Unconventional Ubiquitination in the ER-Associated Degradation Pathway
Source: Int J Proteomics. 2013 Feb 3;2013:857918. doi: 10.1155/2013/857918 (PMC3574754; doi:10.1155/2013/857918)
Supplement: Supplementary file 1 — The supplemental figures included with this manuscript are biochemical analyses that demonstrate that KR-TCRα is modified on an internal, non-lysine residue. In addition, also included is a figure demonstrating that an endogenous, human protein is also ubiquitinated and degraded by the proteasome. [file 857918.f1.pdf]

Figure S1

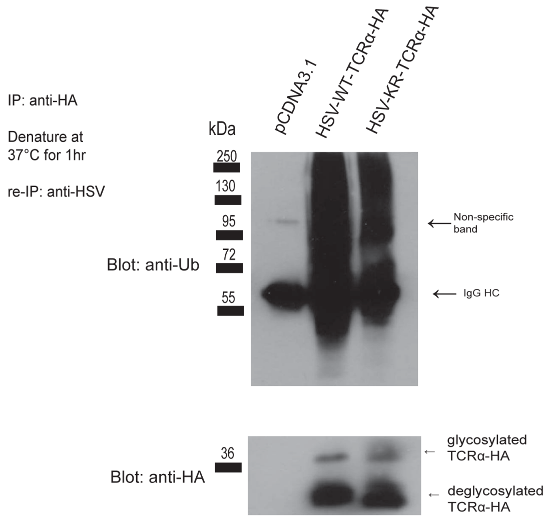

Figure S2A

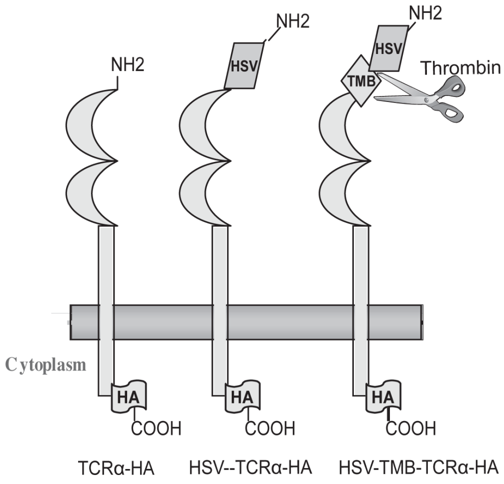

Figure S2B

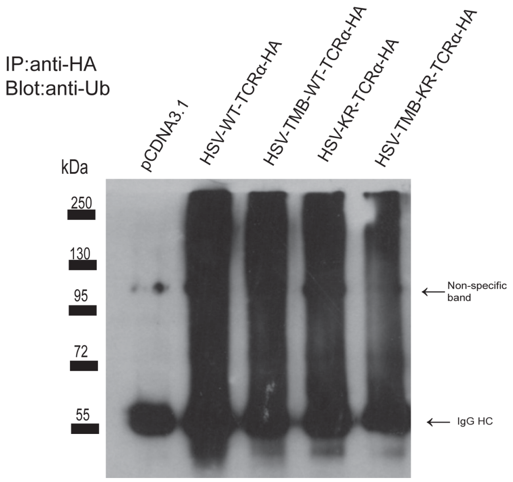

Figure S2C

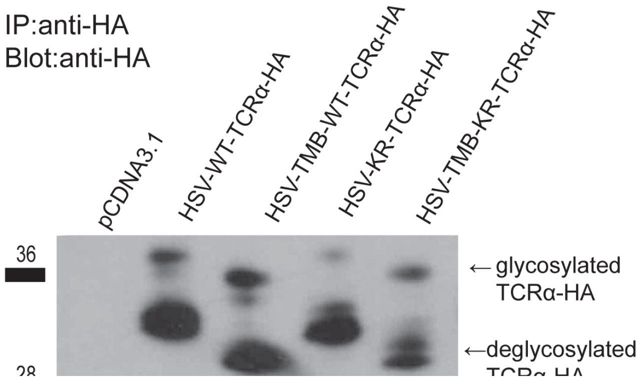

Figure S2D

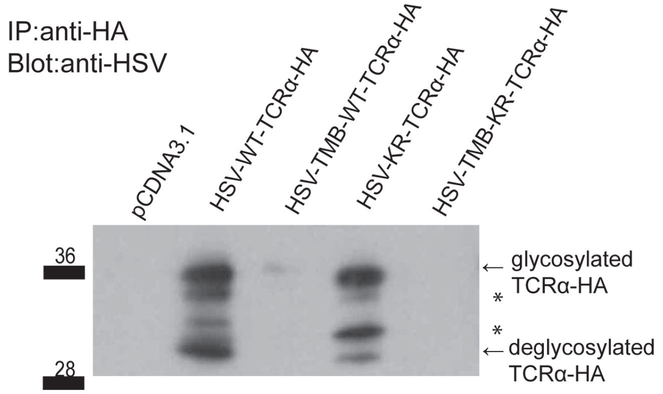

Figure S3A

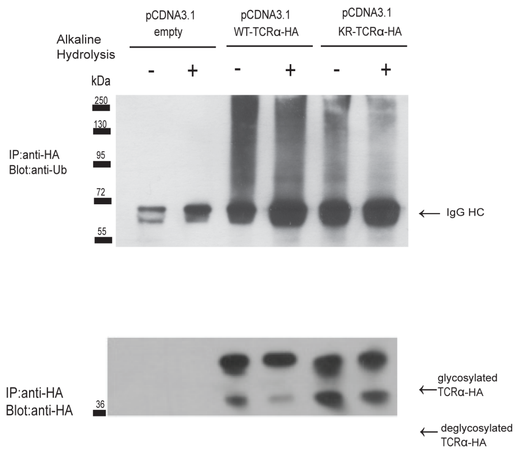

Figure S3B

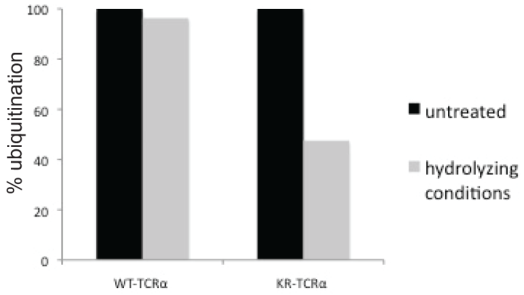

Figure S4

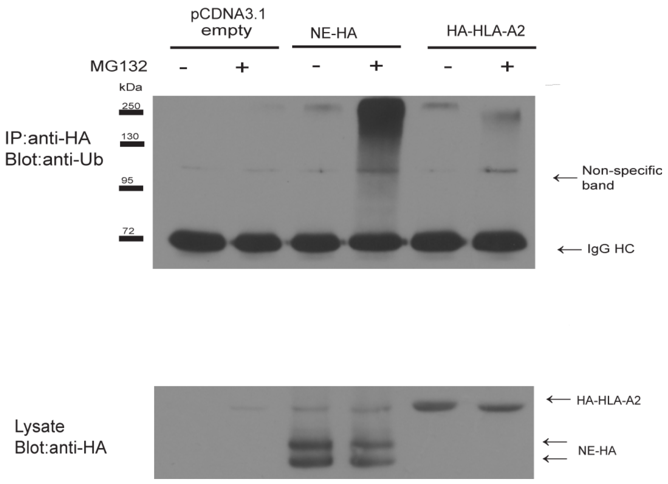

Figure S5

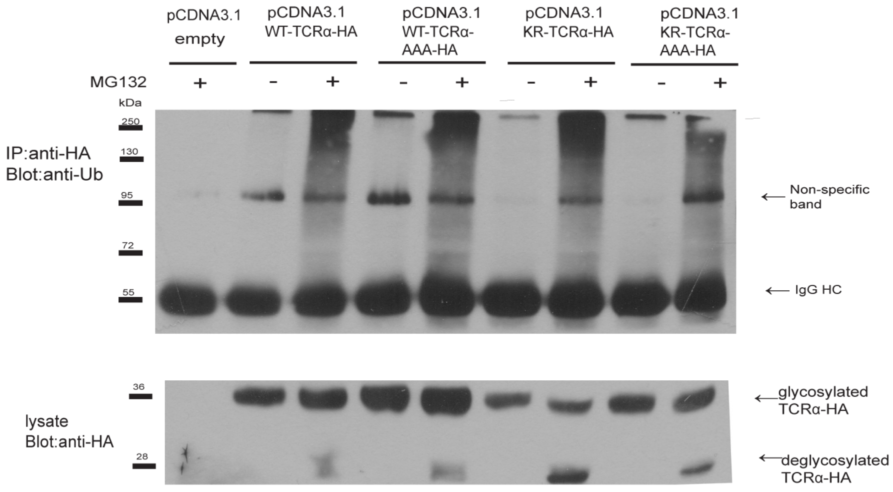
pCDNA3.1
empty
pCDNA3.1
WT-TCRα-HA
pCDNA3.1
WT-TCRα-
AAA-HA
pCDNA3.1
KR-TCRα-HA
pCDNA3.1
KR-TCRα-
AAA-HA
MG132
+
-
+
-
+
-
+
-
+
kDa
250
130
95
72
55
IP:anti-HA
Blot:anti-Ub
Non-specific
band
IgG HC
36
28
lysate
Blot:anti-HA
glycosylated
TCRα-HA
deglycosylated
TCRα-HA

Supplemental Figure Legends

Fig. S1. KR-TCRα is directly modified with ubiquitin.

(A) HEK293T cells were transfected with expression vectors for WT-TCRα or KR-TCRα, treated with MG132 and lysed. Cellular lysates were immunoprecipitated using an anti-HA antibody, denatured for 1hr at 37ºC, and then re-immunoprecipitated with an anti-HSV antibody conjugated to agarose beads. Samples were analyzed by Western blot using an anti-ubiquitin antibody (top panel). Membranes were stripped and reanalyzed using an anti-HA antibody (bottom panel).

Fig. S2. KR-TCRα is not ubiquitinated on the N-terminus.

(A) Schematic diagram of constructs used. (B-D) HEK293T cells were transiently transfected with the different constructs shown. Cells were treated with MG132 before lysis. Cellular lysates were immunoprecipitated using an anti-HA antibody and digested with thrombin. After washing away unbound protein from the beads, samples were analyzed by Western blot using (B) an anti-ubiquitin antibody, (C) an anti-HA antibody or (D) an anti-HSV antibody. Asterisks (*) indicate partially glycosylated forms of TCRα-HA.

Fig. S3. KR-TCRα ubiquitination is sensitive to alkaline hydrolysis.

HEK293T cells were transiently transfected with WT-TCRα and KR-TCRα, incubated with MG132 and then lysed. Lysates were left untreated or subjected to mild alkaline hydrolysis as described in materials and methods. Cellular lysates were then immunoprecipitated using an anti-HA antibody and analyzed by Western blot using (A, left panel) an anti-ubiquitin antibody or (B) an anti-HA antibody. Band intensity was determined using ImageJ, 100% ubiquitination corresponds to the levels of ubiquitination

present in the unhydrolyzed samples for both WT-TCRα and KR-TCRα (A, right panel). These results are representative of five independent experiments.

Fig. S4. An endogenous lysine-less protein, Neutrophil Elastase (NE), is ubiquitinated and degraded by the proteasome.

HEK293T cells were transfected with an empty vector or a vector expressing NE-HA, or HA-HLA-A2. Cells were either left untreated or incubated with MG132 before lysis. Cellular lysates were immunoprecipitated using an anti-HA antibody and the ubiquitination status of NE was analyzed by Western blot analysis using an anti-ubiquitin antibody (top panel). Membranes were stripped and reanalyzed using an anti-HA antibody (bottom panel).

Fig. S5. Thr-262, Ser-267 and Ser-268 are not required for ubiquitination of KR-TCRα

HEK293T cells were transiently transfected with WT-TCRα, WT-TCRα -TSS→AAA, KR-TCRα , or KR-TCRα -TSS→AAA, incubated with MG132 where indicated and then lysed. Lysates were subjected to immunoprecipitation with an anti-HA antibody and examined by SDS-PAGE. The ubiquitination status of these proteins was examined by Western blot analysis (A), and the blots were stripped and reprobed with an anti-HA antibody to verify equal expression levels (B). These constructs were cloned to contain a modified C-terminal HA tag –YPYDVPDYAL* such that the serine residue normally present in HA tags could not be responsible for the ubiquitination status.
